# Long noncoding RNA CASC11 promotes hepatocarcinogenesis and HCC progression through EIF4A3‐mediated E2F1 activation

**DOI:** 10.1002/ctm2.220

**Published:** 2020-11-05

**Authors:** Hang Song, Yan Liu, Xinquan Li, Shuhua Chen, Rongzhang Xie, Dabao Chen, Huawu Gao, Guoquan Wang, Biao Cai, Xiangyu Yang

**Affiliations:** ^1^ School of Integrated Chinese and Western Medicine Anhui University of Chinese Medicine Hefei China; ^2^ Department of Interventional Radiology Beijing Chao‐yang Hospital Affiliated with Capital Medical University Beijing China; ^3^ The Key Laboratory of Bio‐Medical Diagnostics Suzhou Institute of Biomedical Engineering and Technology, Chinese Academy of Sciences Suzhou China; ^4^ Department of laboratory medicine, Yunfu People's Hospital Southern Medical University Yunfu China; ^5^ Anhui Academy of Chinese Medicine Institute of Integrated Chinese and Western Medicine Hefei China; ^6^ Anhui Province Key Laboratory of Chinese Medicinal Formula Anhui University of Chinese Medicine Hefei China

**Keywords:** CASC11, E2F1, EIF4A3, hepatocellular carcinoma, long noncoding RNAs, PD‐L1

## Abstract

**Background:**

Growing evidences have been revealing that long noncoding RNAs are vital factors in oncogenesis and tumor development. Among them, cancer susceptibility candidate 11 (CASC11) has displayed an impressively essential role in various kinds of cancers including hepatocellular carcinoma (HCC). Nevertheless, its role and potential mechanism in HCC still remain to be fully investigated.

**Methods:**

CASC11 expression level was evaluated by real‐time polymerase chain reaction, western blotting, and in situ hybridization staining in HCC patients, and its prognostic effect was analyzed. The role of CASC11 in HCC tumorigenesis and progression was investigated by cell proliferation assay, transwell assay, extracellular acidification rate, western blotting, flow cytometry, and an in vivo xenograft model. The interactions among CASC11, E2F transcription factor 1 (E2F1), and eukaryotic translation initiation factor 4A3 (EIF4A3) were explored by using quantitative reverse transcriptase polymerase chain reaction, western blotting, RNA‐binding protein immunoprecipitation assay, and chromatin immunoprecipitation assays.

**Results:**

Upregulation of CASC11 was confirmed in HCC tissues and associated with poor prognosis. Loss of function assays showed inhibition of CASC11 expression suppressed HCC cells proliferation, mobility, and glucose metabolism and promoted apoptosis. E2F1 expression significantly decreased after inhibition of CASC11. Rescue experiments illustrated that E2F1 overexpression alleviated the suppression of CASC11 inhibition on HCC progression in vitro and in vivo. Mechanistically, CASC11 recruited EIF4A3 to enhance the stability of E2F1 mRNA. CASC11 and E2F1 impacted the activation of the NF‐κB signaling and PI3K/AKT/mTOR pathway and further regulated the expression PD‐L1 that is an important target of immunotherapy. In addition, we identified YY1 could modulate CASC11 expression by binding to its promoter.

**Conclusions:**

Our data revealed that CASC11 promoted the progression of HCC by means of EIF4A3‐mediated E2F1 upregulation, indicating CASC11 is a promising diagnostic biomarker and therapeutic target for HCC.

## Graphical abstract

The current provided cogent evidences verifying that CASC11 was upregulated and correlated with poor prognosis in HCC. Functionally and mechanistically, CASC11 drove HCC progression by recruiting EIF4A3 to regulate E2F1 affecting HCC cells proliferation, cell mobility, apoptosis, and cellular metabolism in vitro and in vivo. Therefore, CASC11 is a promising diagnostic biomarker and therapeutic target for HCC.

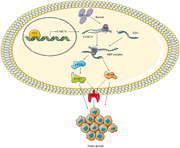

**Abbreviations**
CASC11: cancer susceptibility candidate 11
ChIP: chromatin immunoprecipitation
E2F1: E2F transcription factor 1
ECAR: extracellular acidification rate
EIF4A3: eukaryotic translation initiation factor 4A3
GLUT1: glucose transporter 1
ISH: in situ hybridization
LncRNAs: long noncoding RNAs
MMP: matrix metalloproteinase
NC: negative control
PCNA: proliferating cell nuclear antigen
PD‐L1: programmed cell death 1 ligand 1
RECIST: Response Evaluation Criteria in Solid Tumors
RIP: RNA‐binding protein immunoprecipitation
ROC: receiver operating characteristic
TCGA: The Cancer Genome Atlas

## 1 BACKGROUND

Hepatocellular carcinoma (HCC) is known as one kind of common malignant tumors, ranking the third in cancer‐related deaths.^1^ Although great efforts have been made in developing effective therapies and treatments over the decades, surgery is still the only curative treatment suitable for very few patients in the early stage.^2^, ^3^ Insufficient understanding of underlying mechanism that drives HCC progression hammers the development of HCC treatment. Thus, it is quite crucial to reveal important genes and signal transduction pathways that regulate the development of HCC.

Long noncoding RNAs (LncRNAs) include the noncoding RNAs with more than 200 nucleotides in length.^4^ It has been shown that the occurrence of a variety of diseases is closely related to LncRNAs abnormal expression or dysfunction, including the sequence and spatial structure of abnormalities, abnormal expression levels, abnormal interaction with binding proteins, and so on.^5^ Accumulating evidences have been showing that LncRNAs participate in many important regulatory processes such as cell cycle, chromatin modification, and transcriptional activation and thus are showing great importance in tumor formation, invasion, and metastasis.^6^, ^7^ Recently, cancer susceptibility candidate 11 (CASC11), a kind of LncRNAs, was reported to be a promotor in oncogenesis, tumor development, and metastasis in a distinct variety of malignant tumors including colorectal cancer, gastric cancer, osteosarcoma, ovarian squamous cell carcinoma, and lung cancer.^8^, ^9^, ^10^, ^11^, ^12^ More importantly, several quite new studies, which reported CASC11 could promote proliferation, migration, invasion, epithelial‐mesenchymal transition, and chemoresistance of HCC cells,^13^, ^14^, ^15^ highlighted the impressive significance of CASC11 in HCC, whereas left the clinical signification and underlying mechanism of affecting hepatocarcinogenesis in vitro and in vivo to be fully unfolded.

In the present study, we investigated the expression of CASC11 in HCC as well as its clinical futures of the patients, and found that upregulation of CASC11 was strongly associated with poor prognosis in HCC patients. Next, the effects of CASC11 in HCC cell proliferation, cell mobility, apoptosis, and cellular metabolism were further characterized. Moreover, we identified CASC11 promoted hepatocarcinogenesis by upregulating E2F transcription factor 1 (E2F1), a transcription factor, via recruiting eukaryotic translation initiation factor 4A3 (EIF4A3) in vitro and in vivo. Subsequently, activation of NF‐κB signaling and PI3K/AKT/mTOR pathway along with the expression level of programmed cell death 1 ligand 1 (PD‐L1) was found to be under modulation of both CASC11 and E2F1. In brief, we demonstrate that CACS11 promotes HCC progression by upregulation of E2F1 and their downstream pathways that could be a new therapeutic target for HCC treatment.

## 2 MATERIALS AND METHODS

### 2.1 Tissue samples

The 40 pairs of HCC tissue specimens used in the experiment were collected by biopsy or during operation, placed in liquid nitrogen, and then stored at –80°C until use. Approval by ethics committee of Beijing Chaoyang Hospital was obtained before initiation of the study. Written informed consent were collected from each patient. The effect of treatment was assessed by referring to the criteria of Response Evaluation Criteria in Solid Tumors (RECIST).^16^

### 2.2 Cell culture and transfection

In this experiment, four cell lines of which THHL‐3 and HL‐7702 belong to human liver epithelial cells and HepG2 and SMMC‐7721 represent HCC cell lines were involved. All four cell lines were purchased from the ATCC Cell Biology Collection (Maryland, USA) and stored at –80°C. The Dulbecco's Modified Eagle Medium with 10% fetal bovine serum and 100 U/mL penicillin‐streptomycin was used to culture the cells in a chilled atmosphere of 5% CO_2_ at 37°C.

The CASC11 siRNAs, EIF4A3 siRNA, YY1 siRNA, negative control (NC) siRNAs, E2F1 overexpression plasmids pcDNA‐E2F1, NC plasmids pcDNA‐NC, and luciferase reporter vectors with wild or mutant CASC11 promoter were synthesized by GenePharma (Shanghai, China). The sequences are detailed in Table S1. The lipofectamine 2000 (Invitrogen Life Technologies, USA) was employed to accomplish the cell transfections as previously described.^17^

### 2.3 Quantitative real‐time PCR

RNA extraction from tissues or cells was performed by using TRIzol (Invitrogen, USA), and ABI High‐Capacity cDNA reverse transcription Kit (Thermo Fisher Scientific, Waltham, MA, USA) was used for cDNA extraction. Quantitative real‐time PCR was performed by using the ABI StepOnePlus real‐time PCR system (Applied Biosystem, Foster City, CA, USA), where GAPDH was taken as an internal reference. The primers used are shown in Table S2, and the results are calculated using 2^−ΔΔ^ *^CT^*. The extractions and qRT‐PCR quantification were carried out by following the instructions.

### 2.4 In situ hybridization staining

The paraffin‐embedded HCC and peripheral liver samples were stained to detect CASC11 expression following the instructions of the in situ hybridization (ISH) Kit (BOSTER, Wuhan, China). In brief, the specimens were deparaffined, rehydrated, and then incubated with proteinase at 37°C for 10 min. After a three‐time wash in PBS, the specimens were incubated with hybridization mix at 40°C overnight. Afterward, the sections were blocked by incubation with blocking buffer for 30 min, and then were applied with anti‐DIG for 60 min. A DAB kit (Solarbio, Beijing, China) was used to finally stain the samples; the positive signal was indicated as blue. The probe sequences are listed in Table S3.

Besides, human HCC tissue microarray slides were acquired by purchase from US Biomax (Rockville, MD, USA) and were subjected to ISH staining as described above.

### 2.5 Cell proliferation assay

The Cell Counting Kit‐8 (CCK‐8, Doindo, Japan) assay was performed to access the cell following the manufacturer's protocol. Briefly, 1 × 10^4^ cells were seeded into each well of 96‐well plates. CCK‐8 solutions were added into each well at the particular time point of 4 h at 37°C. The spectrophotometer (Synergy4; BioTek, Winooski, VT, USA) was employed to detect the absorbance at 490 nm.

### 2.6 Colony formation assay

We tested the colony formation ability of the cells as formerly described.^18^ In brief, the cells were trypsinized into single cells and incubated in 6‐well plates for 14 days. Then 0.1% crystal violet and 20% methanol was used to stain the colonies, and the numbers of colonies were counted.

### 2.7 Migration and invasion assays

A Corning costar Transwell chamber was used to perform the cell migration assay. The transfected cells were trypsinized, centrifuged, and resuspended in serum‐free medium. Then, each chamber was filled with a number of 2 × 10^4^ cells, and incubated for 24 h. The upper chamber was drained and subjected to 0.1% crystal violet stain for staining for 15 min at room temperature. After cleaning the crystal violet dye solution, we gently wiped the cells in the upper chamber with a cotton ball, and then dried it naturally. Then, the cells were visualized and counted with microscope at 100 times magnification. After observation, the membranes of chambers were taken out, dissolved in 200 μL of 300 mL/L acetic acid, and placed on a shaker. After shaking for 10 min, 100 μL of each sample was taken and placed in a microplate reader for detection of the absorbance at 570 nm. For the cell invasion assay, similar process was performed except the filters were coated with Matrigel.

### 2.8 Measurement of extracellular acidification rate

We employed the Seahorse Extracellular Flux Analyzer XF96 (Seahorse Bioscience) to measure cellular metabolic level. The manufacturer's instructions were followed during the experiments. We seeded the cells, which were transfected with siRNAs, control siRNAs, EIF2 overexpressing plasmid, and control plasmid, in a XF96‐well plate (8 × 10^3^ cells per well), placed the plate overnight for attachment, and then starved the cells for 24 h with serum deprivation. The cells were sequentially administrated with 10 mM glucose, 1 μM oligomycin, and 80 mM 2‐deoxyglucose during which the extracellular acidification rate (ECAR) was record in an interval of 6 min. Afterward, normalization of ECAR to total protein was performed. Each sample was examined for three times.

### 2.9 Measurement of glucose consumption and lactate production

To measure the intracellular glucose, the cells were lysed and detected by a glucose assay kit (BioVision, San Francisco, CA, USA). For lactate production, we used a lactate assay kit (BioVision) to test the extracellular lactate level in the culture medium. We employed a PK enzyme kit (Solarbio, Beijing, China) to measure the PK activity. Each sample was tested in triplicate.

### 2.10 Northern blot

Total RNA extracts were harvested from tissues and cells by applying the standard TRIZOL methods, and then were electrophoresed through formaldehyde denaturing agarose gel. Then, the separated RNAs were transferred into a NC film. The membrane was subjected to an incubation of hybrid buffer for 2 h. Then, the biotin‐labeled RNA probes were administrated to incubate the membrane. HRP‐conjugated streptavidin (Santa Cruz, CA, USA) was used to detect the biotin signals following the instructions.

### 2.11 Western blot assay

Cells were lysed with a lysate buffer (RIPA buffer, Thermo Fisher Scientific) containing protease inhibitor cocktails (Sigma Chemicals, Poole, UK) to obtain proteins. The protein concentration was quantified by using a BCA protein assay kit (Beyotime, China). The Proteins were electrophoresed in a sodium dodecyl sulfate polyacrylamide gel electrophoresis for separation and transferred onto the polyvinylidene fluoride membrane. Blocking was completed with 5% bovine serum albumin. Afterward, the membranes were incubated with the indicated primary antibodies overnight and then with HRP‐conjugated secondary antibodies (Santa Cruz, CA, USA) at 37°C for 1 h. Detection of proteins used the enhanced chemiluminescence reagent (Santa Cruz, CA, USA). Primary antibodies including proliferating cell nuclear antigen (PCNA), matrix metalloproteinase (MMP)‐2, cleaved caspase 3, PDK1, GLUT1, E2F1, NF‐κB, PD‐L1, PI3K, AKT, β‐actin, and GAPDH were purchased from Abcam (Cambridge, UK).

### 2.12 Target prediction

The interactions between CASC11 and EIF4A3 and between YY1 and CASC11's promoter were predicted by the software of StarBase version 2.0. Transcription factors prediction was finished by browsing the data of PROMO database and TRANSFAC.

### 2.13 RNA sequencing and identification of Different Expressed Genes

The CASC11 expression in The SMMC‐7721 cells was interfered by siRNA‐2 or siRNA‐NC. The cells were lysed to extract the RNA by the Trizol reagent (Invitrogen) at 48 h after being transfected. Then, the RiboMinus Human Transcriptome Isolation Kits (Thermo Fisher Scientific) was employed to eliminate rRNA in the solution. Subsequently, we generated the sequencing libraries by employing the TruSeq Stranded total RNA sample preparation kit (Illumina, San Diego, CA, USA). Then, the number of aligned reads per gene was calculated by HTSEquation (version 0.6.1). The DESeq2 package (http://www.bioconductor.org/packages/release/bioc/html/DESeq2.html) was used to normalize the read counts values from each biological replicate for further analysis.

### 2.14 Subcutaneous tumor model

An approval (#AEEI‐2019‐051) of Committee on the Use and Care on Animals (Capital Medical University, Beijing, China) was obtained before the animal studies. The subcutaneous tumor model of mouse was established as previously reported.^16^ Briefly, a number of 5 × 10^6^ HepG2 cells in 100 μL of PBS were administrated onto the back of each athymic nude mouse. The mice in three groups were administrated with weekly local injection of siRNA‐NC + pcDNA‐NC, siRNA‐2 + pcDNA‐NC, and siRNA‐2 + pcDNA‐EIF4A3, respectively (n = 5). The size of each tumor was measured and recorded every 4 days. The mice were sacrificed 28 days after inoculation. Immediately after execution, the tumor tissues were dissected and weighed.

To establish the lung metastasis model, mice were also divided into three groups (n = 6) such as above subcutaneous model. Each mouse was injected with 2 × 10^6^ transfected cells through tail vein. The mice were euthanatized and dissected at 6 weeks’ postinoculation. The metastatic modules in lung tissues with diameter over 1 mm were sought and counted.

### 2.15 Bioluminescence imaging

The growth of subcutaneous tumors was monitored by bioluminescence imaging as previously reported.^19^ Briefly, we employed a Lumina II imaging system (Caliper Life Sciences). Once a week, the D‐luciferin in the dose of 150 mg/kg was administrated to each mouse via intraperitoneal injection. And then, the mice were anesthetized and imaged 10 min after injection.

### 2.16 RNA‐binding protein immunoprecipitation assay

We carried out the RNA‐binding protein immunoprecipitation (RIP) assay by employing the Magna RIP Kit (Millipore) following the manufacture's instruction. We harvested the transfected cells and used the RIP lysis buffer to turn the cells into lysis. The cell extracts were mixed with magnetic beads and incubated with primary antibodies for 2 h at 4°C. Then purification was performed with TRIzol LS (Life Technology) and the RNAs were quantified by qRT‐PCR analysis.

### 2.17 RNA pull‐down assay

Upregulation of EIF4A3 in SMMC‐7721 cells was achieved by transfection of pcDNA‐EIF4A3 or pcDNA‐NC. The cells were harvested after 48 h since transfection. Then, we lysed the cell with the lysis buffer. The streptavidin‐coated magnetic beads were used to incubate the cell lysates. The incubation lasted for 3 h at 4°C to get the biotin‐coupled RNA complex. Quantification of the RNA‐binding protein mixture was achieved by western blot analysis.

### 2.18 Chromatin immunoprecipitation assay

Chromatin immunoprecipitation assay (ChIP) assay was performed by using the EZ‐CHIPTM chromatin immunoprecipitation kit (Millipore, Germany). In brief, SMMC‐7721 cells were fixed by 1% formaldehyde for 10 min, and then fixation was stopped by adding Glycine into the medium. Next, Lysis Buffer with Protease Inhibitor Cocktail II incubated the cells for 30 min on ice. Afterward, the cells were sonicated and centrifuged. The supernatant was collected and was added with Protein G agarose maintaining for 1 h at 4°C. YY1 antibody and Polymerase II antibody were put into the supernatant. Then, overnight incubation was performed at 4°C. Proteins were digested by proteinase K, and the chromatin was extracted and quantified by qRT‐PCR.

### 2.19 Dual luciferase reporter assay

The luciferase reporter plasmids with wild‐type or mutant CASC11 promoter with either siRNA of YY1 or its control siRNA were transfected into the SMMC‐7721 cells as mentioned above. After 48 h, the cells were tested by Dual‐Luciferase Reporter Assay system (Promega). Normalization of firefly luciferase activity was performed on the basis of Renilla luciferase activity.

### 2.20 Statistical analysis

The experimental data were statistically analyzed using SPSS 17.0. The quantitative data were expressed as mean ± standard error of the mean (SEM), the mean of the two independent samples were compared using the Student's *t* test, and the mean comparison between the multiple groups was analyzed by one‐way analysis of variance. *P*‐values less than .05 were regarded as statistically significant.

## 3 RESULTS

### 3.1 The CASC11 expression was upregulated in the serum and tissues of HCC patients and correlated with prognosis of HCC patients

**FIGURE 1:**
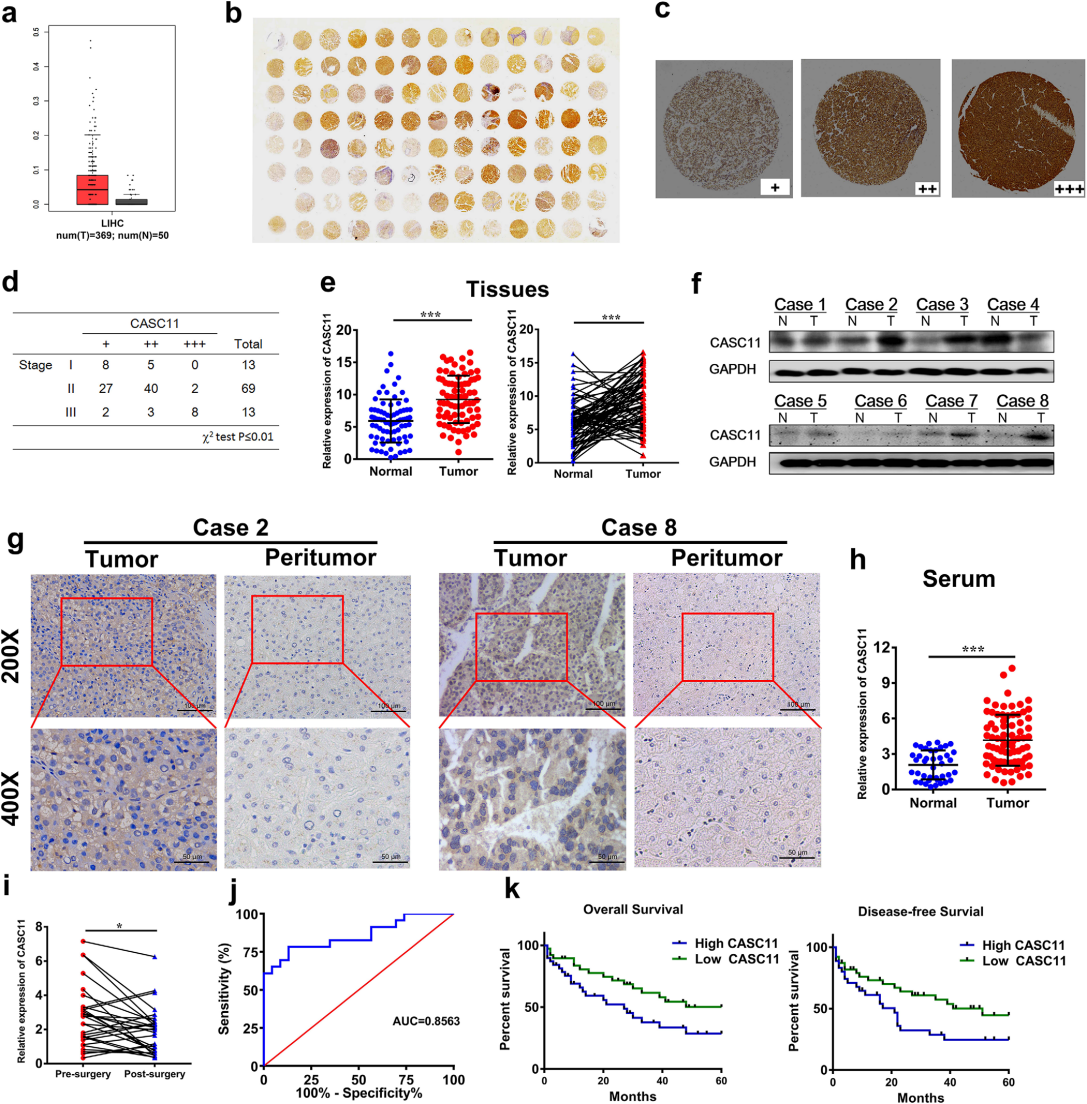
CASC11 expression is increased in the serum and tissues of HCC patients, and the high expression level is negatively associated with prognosis. A, The relative expression level of CASC11 in the HCC tumor and normal tissues according to TCGA database. B, Tissue microarray (TMA) of CASC11. In situ hybridization (ISH) of TMA stained with CASC11 probe. C, Enlarged representative samples expressing different levels of CASC11. D, A chi‐square test was used to analyze the relationship between HCC stage and CASC11 expression. E, The expression of CASC11 in 78 paired HCC tumor and normal tissues was quantified by qRT‐PCR. F, The expression of CASC11 was detected by northern bolting assay. The images of representative patients were shown. G, ISH of the CASC11 in HCC and peritumoral tissues. The representative images of case #2 and case #8 were shown. H, The relative expression of CASC11 in the serum of HCC patients (n = 78) and normal healthy volunteers (n = 40). I, Wilcoxon signed‐rank test of the relative CASC11 expression levels of presurgery and postsurgery. Major of the patients (17/20) showed a decreased CASC11 expression after surgery. The serum CASC11 expression level was detected at 4 weeks after surgery. The patients (n = 29) who were assessed as complete response or partial response according to the RECIST were analyzed. J, Receiver operating characteristic (ROC) curve analysis of serum CASC11 for differentiating the HCC patients and healthy controls. K, Kaplan‐Meier analysis of overall survival and disease‐free survival of HCC patients. The patients were divided into two group by the middle cutoff of CASC11 level. The patients in the higher CASC11 level group demonstrated a more awful prognosis. ^**^ *P* ≤ .01 and ^***^ *P* ≤ .001

Previous researches indicated that the HCC tissues expressed a higher level of CASC11 than normal hepatic tissues.^13^, ^14^, ^15^ We analyzed The Cancer Genome Atlas (TCGA) sequencing data, and it demonstrated an increased expression of CASC11 in HCC tissue comparing with normal tissues (Figure 1A). A tissue microarray including 95 HCC samples was tested by ISH analysis verifying the upregulated expression of CASC11 (Figure 1B). CASC11 overexpression was found to be significantly positively associated with advanced HCC stages (Figures 1C and 1D). Subsequently, in our own cohort, higher expression level of CASC11 was identified in comparison with adjacent normal tissues by qRT‐PCR analysis, northern blotting, and ISH analysis (Figure 1E‐G). Upregulation of CASC11 in the serum of HCC patients instead of normal healthy volunteers was observed (Figure 1H). Moreover, in majority of the patients who were treated with surgery and evaluated as compete response or partial response under RECIST criteria, CASC11 expression in serum was dramatically decreased (Figure 1I). The receiver operating characteristic (ROC) curve analysis in HCC patients and healthy volunteers was carried out. CASC11 demonstrated to be a good candidate for HCC predictors with a high area under the ROC curve (Figure 1J). Further, the patients were divided into two groups by medium cutoff of CASC11 expression in tumor tissues. The patients were divided into two groups by medium cutoff of CASC11 expression level in tumor tissues. The patients with higher CACS11 expression exhibited a larger maximal tumor size than the lower group (Table 1). Furthermore, according to the Kaplan‐Meier survival analysis, the patients with higher expression of CACS11 suffered much more awful overall survival and disease‐free survival (Figure 1K).

**TABLE 1:** CASC11 expression in the tissues of HCC patients measured by qRT‐PCR

Variable	N	High expression	Low expression	*P*‐value
All case	78	39	39	
Age (year)				
>60	44	23	21	.812
≤60	34	16	18	
Gender				
Female	32	15	17	.818
Male	46	24	22	
Maximal tumor size (cm)				
>5.0	41	26	15	.023
≤5.0	37	13	24	
Number of nodules				
>3	30	19	11	.103
≤3	48	20	28	
Lymph node metastasis				
N_0_	42	24	18	.256
N_1_	36	15	21	
Metastasis				
M_0_	51	21	30	.056
M_1_	18	17	9	

### 3.2 Inhibition of CASC11 abated the malignant phenotype of HCC cells in vitro

**FIGURE 2:**
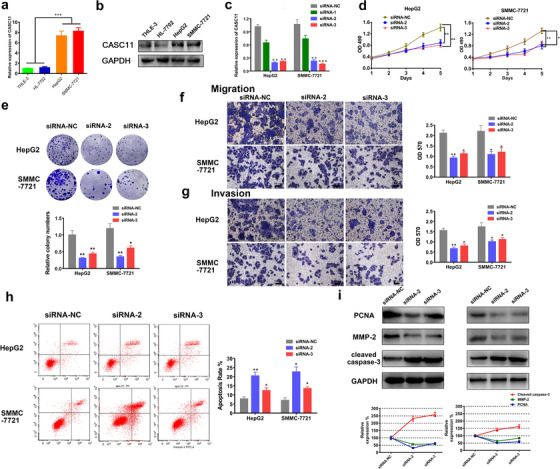
CASC11 silencing suppressed HCC progression in vitro. The relative expression levels of CASC11 in the normal liver cell lines (THLE‐3 and HL‐7702) and HCC cell lines (HepG2 and SMMC‐7721) were detected by qRT‐PCR (A) and northern bolting assay (B). C, The CASC11 expression changed after being treated by siRNAs. The qRT‐PCR was performed 48 h after the HepG2 and SMMC‐7721 cells were transfected by the siRNA‐NC, siRNA‐1, siRNA‐2, and siRNA‐3. D, Growth curve of HepG2 and SMMC‐7721 cells with transfection of siRNA‐2, siRNA3, and siRNA‐NC. The proliferation was measured by CCK‐8 assay. E, Representative bright‐field images and statistics data of colony formation assay. The HepG2 and SMMC‐7721 cells transfected with different siNRAs were seeded on the untreated Petri dishes and incubated for 7 days before staining. Migration (F) and invasion (G) assay for exploring the impaction of CASC11 silencing. The representative images (upper panels) and statistics data (lower panels) were represented. H, Apoptosis rate was measured by flow cytometry. Representative images and statistics data are shown. I, The expressions of PCNA, MMP‐2, and cleaved caspase‐3 with or without CASC11 silencing were detected by using western blotting. ^*^ *P* ≤ .05, ^**^ *P* ≤ .01, and ^***^ *P* ≤ .001

To further explore the impaction of CASC11 on HCC in vitro, CASC11 expression levels in HCC and normal liver cell lines were investigated. Consistent with the clinical results, dramatically higher expression of CASC11 in HCC cell lines HepG2 and SMMC‐7721 was observed in comparison with normal liver cell lines THLE‐3 and HL‐7702 (*P* < .001; Figures 2A and 2B). Then, we developed three siRNAs for interfering the sequence of CASC11. It turned out to be that siRNA‐2 and siRNA‐3 could strongly inhibit the CASC11 expression in both HCC cell lines (Figure 2C). CCK‐8 assay was employed for assessing the cell viability. After interference by siRNA‐2 and siRNA3, the cells grew in a significantly slower rate (Figure 2D). The colony formation assay demonstrated that significantly less colonies were formed by the CASC11‐silenced cells than the control cells (Figure 2E). Further, we performed the transwell assay to detect the ability of migration and invasion. It was shown that less cells permeated through the transwell chambers with or without matrigel after CASC11 inhibition, indicating the impaired ability of migration and invasion in the CASC11‐silenced cells (Figures 2F and 2G). In addition, apoptosis was enhanced after CASC11 silencing (Figure 2H). Because PCNA plays an important role in promoting DNA replication, it has been regarded as a molecular marker for proliferation. The PCNA was quantified. It was shown that the concentration of PCNA in the cells with CASC11 silencing was massively reduced, indicating a depressed status of proliferation (Figure 2I). It has been widely acknowledged that MMPs are essential in regulation of the cell migration.^20^, ^21^ Therefore, the expression of MMP‐2 was also investigated by western blot. The CASC11‐silenced HCC cells held a lower level of MMP‐2, which indicated the impaired ability of migration in accordance with the results of transwell assay (Figure 2I).

**FIGURE 3:**
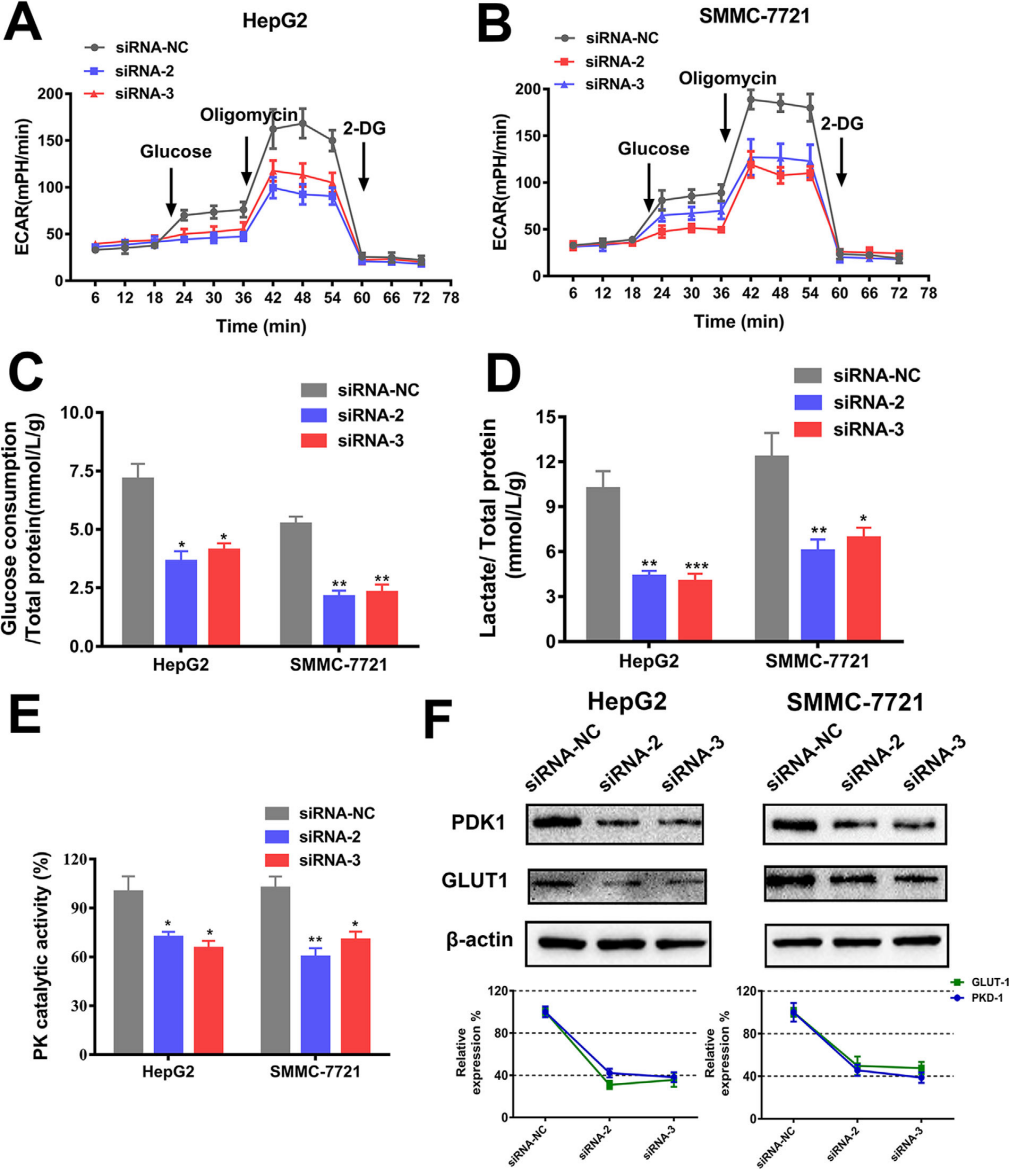
Effects of CASC11 silencing on energy metabolism. A, The ECAR in CASC11‐silenced hepG2 and SMMC‐7721 cells was monitored by the Seahorse XF Cell Energy Phenotype test. Inhibition of CASC11 decreased the glucose uptake (B), lactate levels (C), and PK catalytic activity (D). E, The changes of glucose uptake‐associated proteins (GLUT1 and PDK1) by CASC11 silencing in HCC cells were quantified by western blotting analysis. ^*^ *P* ≤ .05 and ^**^ *P* ≤ .01

As it is known that reprogramming of energy metabolism is one of the essential biological capabilities during the multistep development of human tumors, the impact of CASC11 on cellular metabolism of HCC was also evaluated. ECAR was monitored. The cells with CASC11 silencing exhibited to be in a less energetically active status than the cells transfected with siRNA‐NC (Figure 3A). The glucose consumption rate, the lactate production rate, and PK activity were also significantly lower in the CASC11‐silenced cells in comparison with NC siRNA‐transfected cells (Figure 3B‐D). In addition, the expression of glucose uptake‐associated proteins, glucose transporter 1 (GLUT1) and PDK1, was quantified by western blotting analysis. Downregulated expression of GLUT1 and PDK1 was found when CASC11 was effectively silenced (Figure 3E).

Taken together, CASC11 silencing remarkably abated the malignancy of HCC cells in vitro by powerfully negatively impacting HCC cell proliferation, migration, invasion, and glucose metabolism and enhancing apoptosis, indicating that CASC11 would be an important oncogene for HCC progression.

### 3.3 E2F1 was positively regulated by CASC11

**FIGURE 4:**
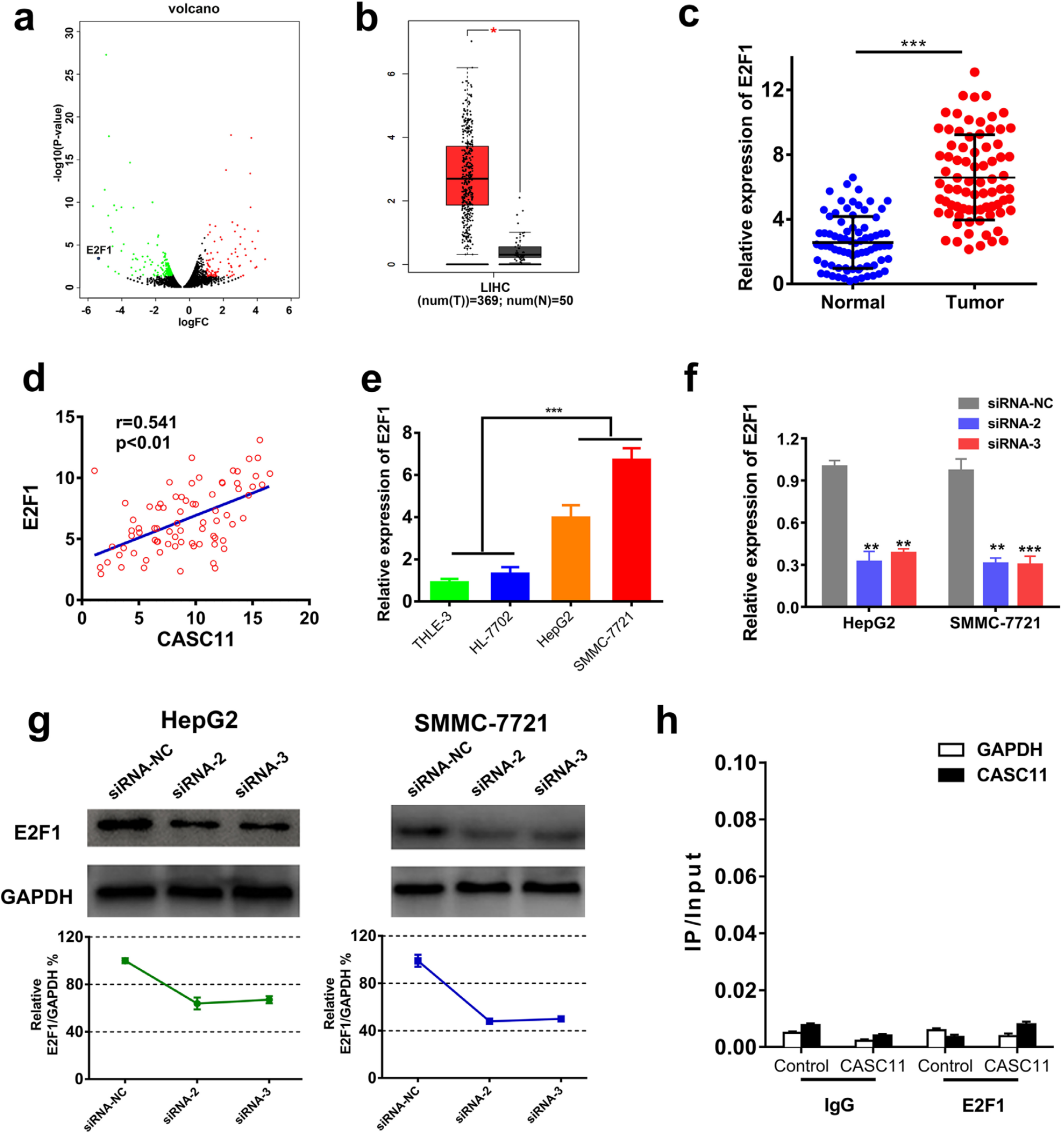
E2F1 was upregulated in HCC and under influenced by CASC11. A, Screening of the mRNAs that were impacted by CASC11 silencing (red spots, remarkably increased mRNAs; green spots, remarkably decreased mRNA). B, The relative expression level of E2F1 in the HCC tumor and normal tissues according to TCGA database. C, The expression of CASC11 in 78 paired HCC tumor and normal tissues was quantified by qRT‐PCR. D, The correlation between E2F1 expression and CASC11 expression in 78 recruited HCC patients. The expression of E2F1 and CASC11 was quantified by qRT‐PCR. E, The relative expression levels of E2F1 in THLE‐3, HL‐7702, HepG2, and SMMC‐7721 were detected by qRT‐PCR. The expression of E2F1 in HCC cells after CASC11 inhibition was tested by qRT‐PCR (F) and western bolting assay (G). H, The pull‐down assay of E2F1 and CASC11. ^**^ *P* ≤ .01 and ^***^ *P* ≤ .001

To further unfold the mechanism of the CASC11 on HCC, underlying downstream targets were sought. We screened the mRNAs that were influenced by CASC11 inhibition. E2F1 came into our sight because it was not only one kind of the most prominently downregulated RNAs after CASC11 silencing (Figure 4A), but also a key factor well‐known for cell cycling as well as HCC tumorigenesis.^22^ We analyzed the TCGA database to access the E2F1 expression profile in HCC patients. TCGA analysis showed that E2F1 was markedly upregulated in the HCC tissues than the normal tissues (Figure 4B). To confirm the results, we also measured the E2F1 expression in the HCC samples of the recruited patients. Constantly, HCC tissues expressed a higher level of E2F1 in comparison with the normal tissues (Figure 4C). The expression of E2F1 was well correlated with the CASC11 expression in the HCC samples (Figure 4D). Additionally, we also confirmed that HCC cell lines expressed a higher level of E2F1 than the normal liver cell lines (Figure 4E). The relationship between CASC11 and E2F1 was further explored. The E2F1 expression level was detected in the cells with or without CASC11 silencing by qRT‐PCR and western bolting analysis. It demonstrated that the CASC11‐silenced cells showed a dramatic decrease of the E2F1 expression compared with NC cells (Figures 4F and 4G). Next, a pull‐down assay was performed to verify whether CASC11 could specifically bind to E2F1. Interestingly, the pull‐down assay disclosed that CASC11 did not catch more E2F1 than NCs (Figure 4H), which means no direct interaction between CASC11 and E2F1 exists. In a word, E2F1 was positively regulated by CASC11 in an indirect manner.

### 3.4 EIF4A3 was recruited by CASC11 to stabilize E2F1

Further explorations were made to identify the mediators by which CASC11 impacts the E2F1.

**FIGURE 5:**
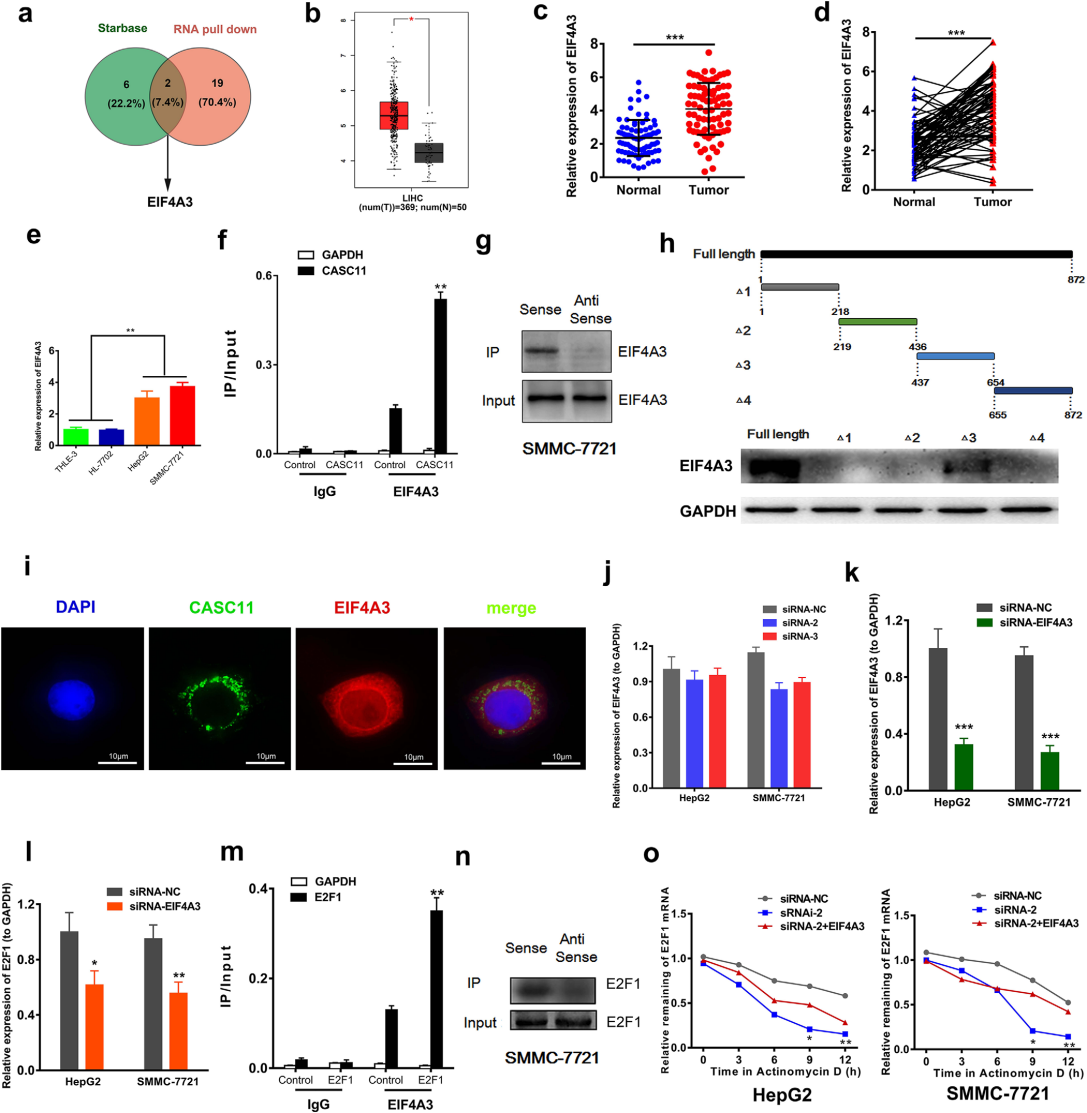
EIF4A3 could interact with CASC11 and E2F1 and contributed to stabilize E2F1. A, Schematic representation of CASC11‐binding targets screening. EIF4A3 belonged to the subcollection of Starbase prediction and pull‐down RNAs. B, The relative expression level of EIF4A3 in the HCC tumor and normal tissues according to TCGA database. C, The relative expression level of EIF4A3 in the HCC tumor and normal tissues (n = 78). Data are represented as mean ± SD. D, Wilcoxon signed‐rank test of the relative EIF4A3 expression levels of HCC tumor and normal tissues. E, The relative expression levels of EIF4A3 in THLE‐3, HL‐7702, HepG2, and SMMC‐7721 cell lines. F, The pull‐down assay of EIF4A3 and CASC11. G, Representative images of western blotting quantification of EIF4A3 that pulled down by CASC11. H, Detection of the binding domain of CASC11. Upper, schematic graph of the fragments of CASC11; lower, western blotting quantification of EIF4A3 bound by each of CASC11 fragments. I, Colocalization of CASC11 and EIF4A3 was verified by immunofluorescence. J, Impact of EIF4A3 expression of CASC11 inhibition was quantified by qRT‐PCR. K, Expression of EIF4A3 was significantly suppressed in HCC cells by siRNA‐EIF4A3 that was detected by qRT‐PCR. L, The expression of E2F1 in HCC cells decreased after EIF4A3 suppression. M, The pull‐down assay of EIF4A3 and E2F1. N, Representative images of western blotting quantification of EIF4A3 that pulled down by E2F1. O, The stability of E2F1 mRNA in HepG2 and SMMC‐7721 was tested by qRT‐PCR after administration of Actinomycin D. ^*^ *P* ≤ .05, ^**^ *P* ≤ 0.01, and ^***^ *P* ≤ 0.001

Target prediction was performed by browsing the Starbase database and RNA pull‐down assay, respectively. EIF4A3 emerged because it was not only a candidate by prediction but also a protein that could be pulled down by CASC11 (Figure 5A). Because EIF4A3 is one kind of RNA‐binding proteins that play an important role in monitoring the mRNA quality and initiating the translation, we believe CASC11 should work together with it to promote HCC progression. The expression of EIF4A3 in HCC was profiled. The results of TCGA data, recruited patients as well as the HCC cells correspondingly illustrated the upregulation of EIF4A3 (Figure 5B‐E). The abnormal expression of EIF4A3 implied it would be essential in the HCC tumorigenesis.

The role of EIF4A3 was investigated mechanically and functionally. RIP assay and RNA pull down assay were performed to verify whether CASC11 could specifically bind to EIF4A3. It was shown that a strikingly larger amount of EIF4A3 was pulled down after CASC11 overexpression in SMMC‐7721 cells (Figures 5F and 5G). Besides, colocalization of CASC11 and EIF4A3 in confocal assay also supported specific binding between them (Figure 5I). To further determine the binding site, a series of deletion mutants of CASC11 were prepared to locate the EIF4A3‐binding regions. The results of immunoblot demonstrated that CASC11 mutantsΔ3 most efficiently bounded to EIF4A3, whereas other mutants did not exhibit any noteworthy binding capacity (Figure 5H), which means nucleotides 437–654 of CASC11 are the essential part of binding to EIF4A3. To figure out whether EIF4A3 expression was under regulation of CASC11, the expression of EIF4A3 was detected in the CASC11‐silenced HCC cells. No significant expression change was observed (Figure 5J), implying that EIF4A3 would be recruited by CASC11 to regulate downstream targets. A small interfering RNA for EIF4A3 inhibition was developed to determinate the relationship between EIF4A3 and E2F1 (Figure 5K). We found E2F1 expression decreased after EIF4A3 expression was interfered (Figure 5L). The interaction between E2F1 and EIF4A3 was evaluated by RIP assay and RNA pull down assay, and an essential binding between them was discovered (Figures 5M and 5N). As known, RNA‐binding proteins (RBP) can interact with LncRNAs to change the stability of mRNAs. Therefore, we evaluated the stability of E2F1 mRNA in HCC cells subjected to actinomycin D after transfections. CASC11 silencing dramatically decreased the stability of E2F1 mRNA, whereas its stability was relatively more fortified with both CASC11 knockdown and E2F1 overexpression (Figure 5O). In together, these results demonstrated that CASC11 recruited EIF4A3 to regulate the E2F1 expression by enhancing the stability of E2F1 mRNA.

### 3.5 Overexpression of E2F1 substantially reversed the suppressive effects of CASC11 inhibition on HCC in vitro and in vivo

**FIGURE 6:**
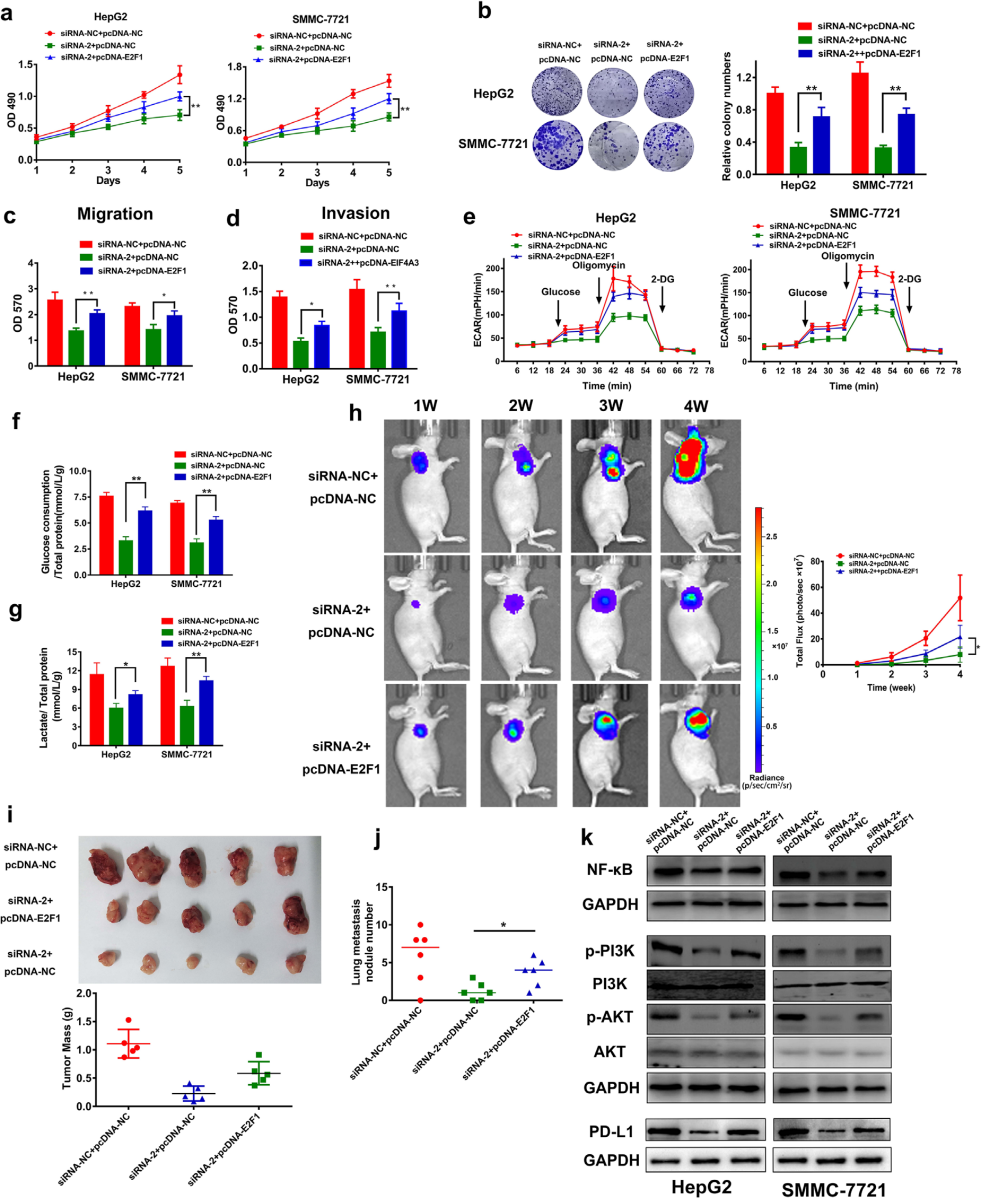
Overexpression of E2F1 substantially reversed the suppression of CASC11 inhibition on HCC in vitro and in vivo. A, Growth curves of HepG2 and sMMC‐7721 cells with transfection of siRNA‐NC + pcDNA‐NC or siRNA‐2 + pcDNA‐NC or siRNA‐2 + pcDNA‐E2F1. B, Colony formation assay of the HepG2 and SMMC‐7721 cells with CASC11 silencing and E2F1 rescue. C and D, Transwell assays with or without Matrigel (for invasion) were performed to evaluate the changes of migration and invasion upon the rescue by E2F1 overexpression. E, The ECAR in CASC11‐silenced hepG2 and SMMC‐7721 cells with or without E2F1 rescue was monitored. The glucose uptake (F) and lactate levels (G) were tested in the cells with CASC11 inhibition and E2F1 rescue. H, Bioluminescent imaging of the subcutaneous HepG2 tumor model. The mice were intratumoral transfected with siRNA‐NC + pcDNA‐NC or siRNA‐2 + pcDNA‐NC or siRNA‐2 + pcDNA‐E2F1 and were imaged once a week. Representative images and statistic data of bioluminescent activity were shown. I, Photograph (upper) and the weight (lower) of dissected tumors at 4 weeks after inoculation. J, The numbers of metastatic nodules in a lung metastasis mouse model. The mice were sacrificed 5 weeks after tail vein injection of HepG2 cells and metastatic nodules with diameter ≥ 1.0 mm were counted. K, Western bolt analysis of the important proteins of NF‐κB signaling (upper), PI3K/AKT/mTOR (middle) pathways, and PD‐L1 (lower). ^*^ *P* ≤ .05, ^**^ *P* ≤ .01, and ^***^ *P* ≤ .001

To further validate if E2F1 is a fundamentally functional downstream target of CASC11, the following experiments were performed. First of all, we overexpressed E2F1 in HCC cells to observe the change of proliferation. It was detected that E2F1 overexpression rescued the inhibited proliferation of HCC cells that was induced by CASC11 knockdown (Figure 6A). The colony formation assay was also employed. Compared with the cells with only CASC11 inhibition, the cells with both CASC11 inhibition and E2F1 overexpression developed much more colonies (Figure 6B). Cell mobility assays also illustrated E2F1 overexpression relived the suppression by CASC11 inhibition (Figures 6C and 6D). We also tested the energy metabolism. As expected, the ECAR, glucose consumption rate, and the lactate production rate assays consistently revealed that E2F1 overexpression solidly reversed the status of low‐energy metabolism in CASC11‐silenced HCC cells (Figure 6E‐G).

Subsequently, we utilized a subcutaneous model to evaluate the effects of CASC11 and E2F1 in vivo. HepG2 cells with stably luciferase expression were injected into the nude mice (n = 6). The growth curve was recorded by measuring the luciferase activity weekly. Constant with the results in vitro, the tumors grew significantly slower after CASC11 inhibition, whereas the suppression was apparently diminished in the tumors with E2F1 overexpression (Figures 6H and 6I). To estimate the influence in metastatic potential, a lung metastasis mouse model was established by tail vein injection of HepG2 cells. The mice with injection of CASC11‐silenced and E2F1‐overexpressed cells bore a quite larger numbers of metastatic nodules than the mice treated with CASC11 knockdown only cells (Figure 6J).

E2F1 regulates the cellular signals including NF‐κB and PI3K/AKT/mTOR.^23^ Meanwhile, these pathways are displayed to be fundamental in HCC development.^24^, ^25^ Thus we investigated the impact of CASC11 inhibition and E2F1 overexpression on these pathways. The results illustrated that NF‐κB and PI3K/AKT/mTOR pathways were both substantially inhibited after CASC11 silencing, whereas the suppression was remarkably relieved upon overexpression of E2F1 (Figure 6K). Particularly, PD‐L1 was reported to be driven by the activation of these signaling pathways.^26^, ^27^ Therefore, we examined the PD‐L1 expression changes by western blotting assay and similar patterns were found with CASC11 inhibition and rescue of E2F1 overexpression (Figure 6K). So it is indicated that the activation of NF‐κB and PI3K/AKT/mTOR pathway as well as PD‐L1 expression was regulated by CASC11 via modulating E2F1.

### 3.6 CASC11 expression was regulated by YY1

**FIGURE 7:**
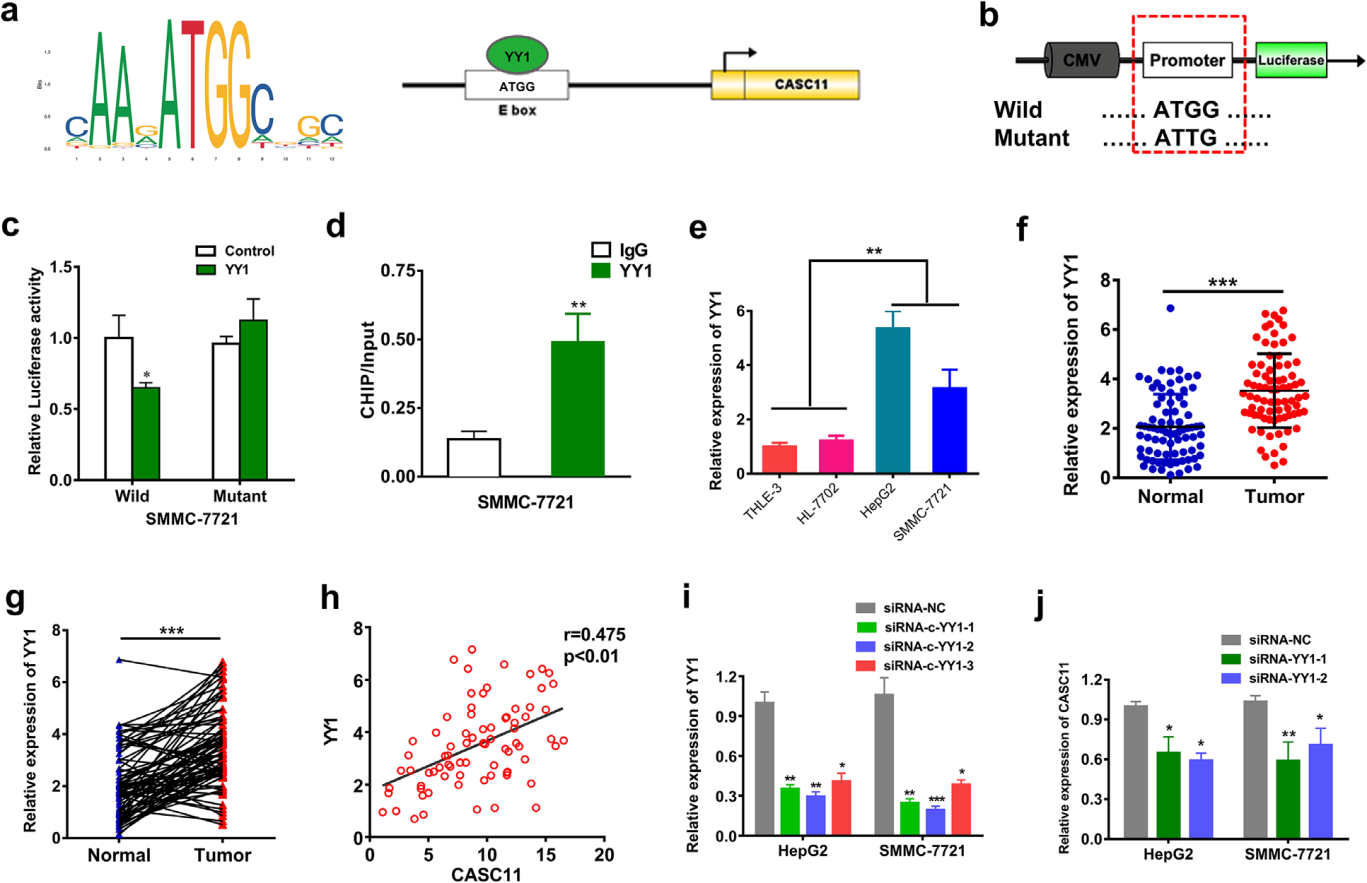
YY1 could target CASC11's promoter to regulate its expression. A, A diagram showed the sequences of promising binding site of YY1 in the promoter of CASC11. B, Schematic display of the luciferase reporter gene with wild or mutant CASC11 promoter. C, The luciferase activities of the cells with wild or mutant CASC11 promoter after transfected with YY1 inhibitor. D, ChIP assay of YY1 and CASC11. E, The relative expression levels of YY1 in THLE‐3, HL‐7702, HepG2, and SMMC‐7721 cell lines. F, The relative expression level of YY1 in the HCC tumor and normal tissues (n = 78). Data are represented as mean ± SD. G, Wilcoxon signed‐rank test of the relative YY1 expression levels of HCC tumor and normal tissues. H, YY1 expression was positively correlated with CASC11 expression in HCC. I, YY1 expression levels decreased when inhibited by the siRNA‐YY1s. J, The expression of CASC11 decreased with YY1 silencing. ^*^ *P* ≤ .05, ^**^ *P* ≤ .01, and ^***^ *P* ≤ .001

We further explored the mechanism of regulating CASC11 expression. By target prediction, YY1 demonstrated a great potential of binding to the promoter of CASC11 (Figure 7A). To investigate if YY1 could interact with the CASC11's promoter, a plasmid with wild or mutant CASC11's promoter and luciferase reporter gene was built and transfected into the SMMC‐7721 cells. (Figure 7B). When the YY1 expression was silenced, the luciferase activity of the cells with wild CASC11's promoter was substantially decreased, whereas no significant changes were found in the cells with mutant type (Figure 7C). Next, ChIP assay was performed and the results showed that YY1 could directly bind to the CASC11's promoter (Figure 7D). The expression level of YY1 in HCC was also tested. And it was found that YY1 was notably upregulated in both HCC cell lines and tumor tissues that specifically correlated with the expression of CASC11 (Figure 7E‐H). In addition, we observed that YY1 silencing could functionally decrease the expression of CASC11 (Figures 7I and 7J). In all, our data disclosed that YY1 could target CASC11's promoter to regulate its expression.

## 4 DISCUSSION

HCC is one of the worldwide life‐threatening malignant tumors, which is in urgent need for expanding the understanding of underlying mechanism to support therapy development. LncRNAs abnormal expression or dysfunction was associated with many biological and pathological processes and was identified to play a vital role in many kinds of tumors.^4^, ^28^, ^29^ LncRNA CASC11 has shown to be an important oncogene in many kinds of cancers such as colorectal cancer, gastric cancer, osteosarcoma, ovarian squamous cell carcinoma, and lung cancer. As yet, the functional roles of CASC11 and its mechanism in HCC still have not been fully clarified. In this research, the data showed that CASC11 was upregulated in HCC tissues and cells lines, and its high expression was associated with more advanced tumor progression and poorer prognosis. When CASC11 was effectively interfered by siRNAs, the proliferation, colony formation, migration, invasion, antiapoptosis, and glucose metabolism of HCC cell lines were dramatically inhibited. In a word, it is identified that CASC11 served as an important oncogene in the development of HCC.

It should be mentioned that Nan et al's research has reported that the expression of CASC11 in HCC was upregulated and associated with poor prognosis of the HCC patients.^15^ According to their study, CASC11 might associate with miR‐188‐5p to modulate HCC cells proliferation. Besides, it was reported by Han et al that STAT3 could regulate CASC11 to impact HCC cell migration, invasion, and epithelial‐mesenchymal transition.^13^ Nevertheless, more in‐depth studies were needed to fully exploit more integrated functional roles and to illuminate further underlying regulatory mechanism of CASC11 on HCC. In comparison with former studies, the current study focused on the upstream and downstream interacting targets of CASC11 and in‐depth investigation was performed relatively.

E2F1, belonging to the E2F transcription factor family, is acknowledged as an important initiator for cell to enter into the S phase. Its emerging role in the tumorigenesis and progression of many types of malignant tumors including HCC was recognized in recent years.^30^, ^31^ However, its function in regulating the HCC progression still needs to be fully illustrated. By screening the mRNAs that were influenced by CASC11 knockdown, we were aware of that E2F1 gene expression was highly related to CASC11. We also found E2F1 was upregulated in the samples of TCGA and recruited HCC patients. Nevertheless, no direct binding between CASC11 and E2F1 was detected. Therefore, E2F1 should be regulated by CASC11 via other mechanisms.

Previous studies revealed LncRNAs can recruit RBPs to regulate the downstream genes by changing the stability of their mRNAs.^32^, ^33^, ^34^, ^35^ Through dual screening of target prediction and RNA pull down assay, we identified EIF4A3, a vital exon junction complex component that participates in the development of secondary structure of mRNA in the 5′UTR region and promotes the initiation of translation of proteins.^36^, ^37^ We confirmed CASC11 could specially bind to EIF4A3, and the binding site was located in fragment of nucleotides 437‐654 of CASC11. Further investigation disclosed that EIF4A3 could bind to E2F1 and affect the expression of E2F1. Moreover, the stability of E2F1 mRNA was found to be under‐regulated by CASC11/EIF4A3 axis. Hence, we revealed that CASC11 employed EIF4A3 to stabilize the E2F1 for further regulating HCC progression.

What's more, this study expanded the knowledge about the upstream and downstream of CASC11/E2F1 regulatory associations. It is revealed that NF‐κB signaling and PI3K/AKT/mTOR pathway were modulated by both CASC11 and EIF4A3. Immunotherapy brought new hopes in anticancer fights with a series of kinds of tumors. However, unresponsiveness of checkpoints blockers exists in a relatively large portion of patients due to insufficient and heterogeneous expression of PD‐L1 in tumor.^26^, ^38^ The results illustrated that PD‐L1 decreased after CASC11 inhibition. This indicated that PD‐L1‐induced immune escape might be an important way for CASC11 to promote HCC progression. Consequently, CASC11 might be a potential target for immunotherapy. What's more, we found YY1 could regulate the CASC11 expression by binding to the promoter of CASC11.

**FIGURE 8:**
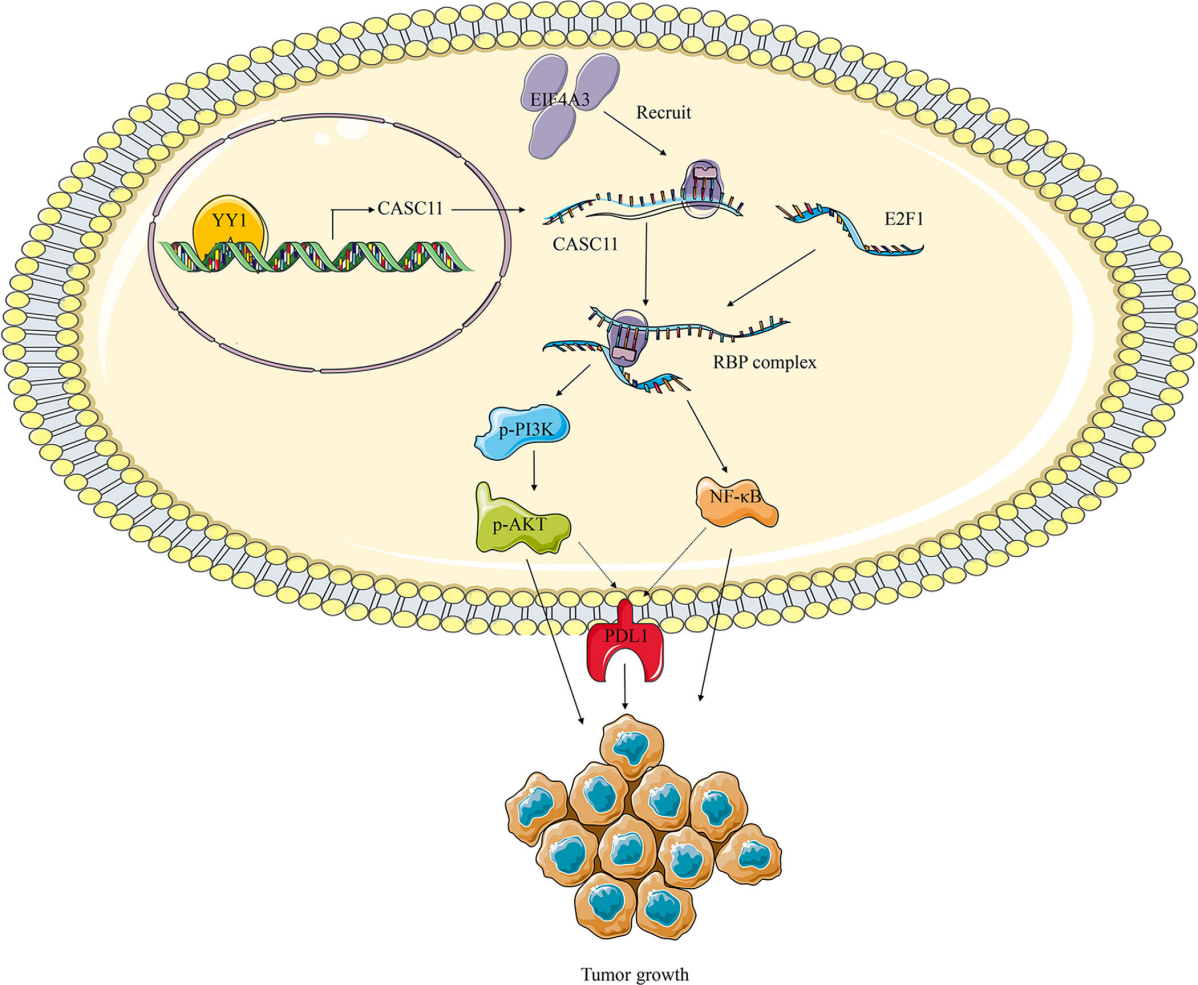
Schematic diagram of mechanism on this research. CASC11, modulated by YY1, recruits EIF4A3 and further regulates E2F1 and then further impacts the activation of NF‐κB signaling and PI3K/AKT/mTOR pathway in HCC and PD‐L1 expression

In summary, we report CASC11, which was modulated by YY1, plays a crucial role in promoting HCC progression by recruiting EIF4A3 to affect E2F1 and then further impacting the activation of NF‐κB signaling and PI3K/AKT/mTOR pathway (Figure 8).

## 5 CONCLUSION

Our study provided informative and cogent evidences verifying that CASC11 was upregulated and correlated with poor prognosis in HCC. For the first time, it was functionally and mechanistically demonstrated that CASC11 drove HCC progression by recruiting EIF4A3 to regulate E2F1 affecting HCC cells proliferation, cell mobility, apoptosis, and cellular metabolism in vitro and in vivo. Therefore, CASC11 is a promising diagnostic biomarker and therapeutic target for HCC.

## ETHICS APPROVAL AND CONSENT TO PARTICIPATE

This project was approved by ethics committee of Beijing Chaoyang Hospital. Details are in Section 2.1.

## CONSENT FOR PUBLICATION

Each author approved the manuscript before submission for publication.

## AVAILABILITY OF DATA AND MATERIAL

The data used to support the findings of this study are available from the corresponding author upon request.

## CONFLICT OF INTEREST

The authors declare no conflict of interest.

## AUTHOR CONTRIBUTIONS

XY and BC proposed the experimental design. HS, YL, and XL executed the experiments. HS, SC, and RX helped with data interpretation and bioinformatics analysis. HS, HG, and GW composed the manuscript. The final manuscript was reviewed and approved by all authors before submission.

## Supporting information

TableS1‐S3
