# Supplementary material for: Long noncoding RNA CASC11 promotes hepatocarcinogenesis and HCC progression through EIF4A3‐mediated E2F1 activation
Source: Clin Transl Med. 2020 Nov 5;10(7):e220. doi: 10.1002/ctm2.220 (PMC7643871; doi:10.1002/ctm2.220)
Supplement: Supplementary file 1 — TableS1‐S3 [file CTM2-10-e220-s001.docx]

**Table S1. Sequences of siRNAs.**

| **Probe** | **Sequence (5’-3’)** |
| --- | --- |
| CASC11 siRNA-1 | AGUAACUCCUCUUUCUUCGGA |
| CASC11 siRNA-2 | GCCCACAUCAAGCCUUCAU |
| CASC11 siRNA-3 | GGAACUCACCAGCCAAGUU |
| YY1 siRNA-1 | GGCAGAAUUUGCUAGAAUG |
| YY1 siRNA-2 | GCUCCAAGAACAAUAGCUUGC |
| YY1 siRNA-3 | GCAAGAAGAGUUACCUCAG |
| EIF4A3 siRNA | AAGCAGCAGAUCAGUGGGAUGAG |
| Negative Control (NC) | UUCUCCGAACGUGUCACGU |

**Table S2. Sequences of primers.**

| **primer** | **Sequence (5’-3’)** |
| --- | --- |
| CASC11 | F: GCTGCAGAAGGTCCGAAGAA |
|  | R: TTCACCACGTCCAGTTGCTT |
| E2F1 | F: ACGCTATGAGACCTCACTGAA |
|  | R: TCCTGGGTCAACCCCTCAAG |
| EIF4A3 | F: GGGGCATCTACGCTTACGG |
|  | R: GCGATGACATCTCTCCCTTTGA |
| YY1 | F: ACGGCTTCGAGGATCAGATTC |
|  | R: TGACCAGCGTTTGTTCAATGT |
| GAPDH | F: AAATCCCATCACCATCTTCCAG |
|  | R: TGATGACCCTTTTGGCTCCC |

**Table S3. Sequences of ISH probes.**

| **Probe** | **Sequence (5’-3’)** |
| --- | --- |
| CASC11 | GGCTTCAGTCTCACCCCTAAGTTCGCT |
| Negative Control (NC) | GTGTAACACGTCTATACGCCCA |
